# Supplementary material for: Autophagy regulates inflammatory programmed cell death via turnover of RHIM-domain proteins
Source: eLife. 2019 Jul 9;8:e44452. doi: 10.7554/eLife.44452 (PMC6615860; doi:10.7554/eLife.44452)
Supplement: Supplementary file 1. [file elife-44452-supp1.docx]

**Supplementary file 1**

| **Genotype** | **Number Expected** | **Actual Number** |
| --- | --- | --- |
| *Ripk1^+/+^* | 17.25 (25%) | 20 (29%) |
| *Ripk1^RHIM/+^* | 34.5 (50%) | 49 (71%) |
| *Ripk1^RHIM/RHIM^* | 17.25 (25%) | 0 |

Number of pups from intercrossing *Ripk1^RHIM/+^ Zbp1^fl/fl^* mice.
