# Supplementary material for: Autophagy regulates inflammatory programmed cell death via turnover of RHIM-domain proteins
Source: eLife. 2019 Jul 9;8:e44452. doi: 10.7554/eLife.44452 (PMC6615860; doi:10.7554/eLife.44452)
Supplement: Supplementary file 2. [file elife-44452-supp2.docx]

**Supplementary file 2**

| **Gene** | **20mer** |
| --- | --- |
| *Atg14L* | TGTGCAACACCACTCGCCGG |
| *Atg14L* | GCAAGTCAACCGCCGGCGAG |
| *Atg16l1* | ACTGCACAAGAAGCGTGGGG |
| *Atg16l1* | GGGTCTGGTTGGCTACCTCG |
| *eGFP* | GGTGGTGCAGATGAACTTCA |
| *eGFP* | GGCATCGACTTCAAGGAGGA |
| *Gsdmd* | AGGTTGACACATGAATAACG |
| *Gsdmd* | AAGTCTCTGATGTCGTCGAT |
| *Mlkl* | GACTTCATCAAAACGGCCCA |
| *Mlkl* | GCACACGGTTTCCTAGACGC |
| *Ptprc* | TATTAATTCTTACCATCACT |
| *Ptprc1* | CAAACACCTACACCCAGTGA |
| *Rb1cc1* | AGAGTGTGTACTTACAGCGC |
| *Rb1cc1* | CAGGTTCTGGTGGTCAATGG |
| *Ripk3* | GGAACCGCTGACGCACCAGT |
| *Ripk3* | CGGACACGAAGTCCCACTGG |
| *Rubcn* | TCTTACCTTCTCCACGTGAA |
| *Rubcn* | TCTTACCTTCTCCACGTGAA |
| *Ticam1* | TCTGGTGTGTCAATGGGACG |
| *Ticam1* | CAAGCTATGTAACACACCGC |
| *Zbp1* | TGAGCTATGACGGACAGACG |
| *Zbp1* | CAGGTGTTGAGCGATGACGG |
| *Nlrp3* | ATGAACTCCTGACCATCGGC |
| *Nlrp3* | GTTCTTTATCCACTGCCGAG |
| *Pycard* | GTGCAACTGCGAGAAGGCTA |
| *Pycard* | ACAAGTTTTCAAGAGCGTCC |
